# Subcommittee on the taxonomy of *Halobacteriaceae* and Subcommittee on the taxonomy of *Halomonadaceae*: Minutes of the joint open meeting, 24 June 2013, Storrs, Connecticut, USA

**DOI:** 10.1099/ijs.0.055988-0

**Published:** 2013-09

**Authors:** Aharon Oren, Antonio Ventosa

## 

### Minute 1. Call to order.

The meeting was held at the University of Connecticut, Storrs. The chairman, Dr A. Ventosa, opened the meeting at 18 : 50.

### Minute 2. Record of attendance.

The subcommittee members present were Drs A. Ventosa (Chairman, Subcommittee on the Taxonomy of *Halobacteriaceae* and Subcommittee on the taxonomy of *Halomonadaceae*), A. Oren (Secretary, Subcommittee on the taxonomy of *Halobacteriaceae*), R. R. de la Haba (Spain), M. R. Mormile, R. T. Papke, R. Montalvo-Rodriguez, F. Rodríguez-Valera and R. H. Vreeland. Apologies were received from D. R. Arahal, V. Bejar, R. R. Colwell, M. L. Dyall-Smith, A. Gambacorta, M. Kamekura, S.-J. Liu, Y. Ma, E. Quesada, H. Stan-Lotter and B. J. Tindall.

In addition, the meeting was attended by P. Corral (Spain), K. Dolas (USA), A. B. Fernandez (Spain), J. P. Gogarten (USA), S. P. Kanekar (India), M. J. Leon (Spain), C. López-Hermoso (Spain), T. McGenity (UK), L. Pašić (Slovenia), J. E. Santiago-Cenea (Puerto Rico), E. L. Tosado-Rodriguez (Puerto Rico), J. Wiegel (USA) and X.-W. Xu (China).

### Minute 3. Appointment of secretary.

A. Oren (Secretary, Subcommittee on the taxonomy of *Halobacteriaceae*) was appointed secretary of this joint subcommittee meeting.

### Minute 4. Approval of agenda.

The agenda of the meeting was approved.

### Minute 5. In memoriam Carol D. Litchfield.

Professor Carol D. Litchfield, who was a member of the Subcommittee on the taxonomy of *Halobacteriaceae* from 1996 and was a founding member of the Subcommittee on the taxonomy of *Halomonadaceae*, passed away in April 2012. R. H. Vreeland presented a short obituary in her memory. The species *Halohasta litchfieldiae* [Mou *et al.*, *Extremophiles* **16** (2012) 895–901] was named in Carol’s honour.

### Minute 6. Report of the chairman.

A. Ventosa explained about the work of the subcommittees and stressed the importance of the participation of young scientists in the subcommittees’ work.

### Minute 7. New taxa within the family *Halobacteriaceae*.

The new names associated with taxa within the family were reviewed by A. Oren. As of June 2013, the family *Halobacteriaceae* contained 40 genera and 144 species whose names have standing in nomenclature (genus name, recommended three-letter abbreviation and number of species): *Halobacterium* (*Hbt*. 3), *Haladaptatus* (*Hap.* 3), *Halalkalicoccus* (*Hac.* 2), *Halarchaeum* (*Hla.* 2), *Halarchaeobius* (*Hab*. 1), *Haloarcula* (*Har.* 9), *Halobaculum* (*Hbl.* 2), *Halobellus* (*Hbs*. 3), *Halobiforma* (*Hbf.* 3), *Halococcus* (*Hcc.* 7), *Haloferax* (*Hfx.* 11), *Halogeometricum* (*Hgm.* 2), *Halogranum* (*Hgn.* 4), *Halolamina* (*Hlm.* 1), *Halomarina* (*Hmr.* 1), *Halomicrobium* (*Hmc.* 3), *Halonotius* (*Hns.* 1), *Halopelagius* (*Hpl.* 2), *Halopenitus* (*Hpt*. 1), *Halopiger* (*Hpg.* 2), *Haloplanus* (*Hpn.* 3), *Haloquadratum* (*Hqr.* 1), *Halorhabdus* (*Hrd.* 2), *Halorientalis* (*Hos.* 1), *Halorubrum* (*Hrr.* 25), *Halosarcina* (*Hsn*. 2), *Halosimplex* (*Hsx.* 1), *Halostagnicola* (*Hst.* 3), *Haloterrigena* (*Htg.* 9), *Halovenus* (*Hvn*. 1), *Halovivax* (*Hvx.* 2), *Natrialba* (*Nab.* 6), *Natrinema* (*Nnm. 7*), *Natronoarchaeum* (*Nac.* 2), *Natronobacterium* (*Nbt.* 1), *Natronococcus* (*Ncc.* 4), *Natronolimnobius* (*Nln.* 2), *Natronomonas* (*Nmn.* 2), *Natronorubrum* (*Nrr.* 6), *Salarchaeum* (*Sar.* 1).

An updated list of taxa will be uploaded on the home page of the subcommittee on the ICSP website.

Reports on the following new taxa and reclassification of existing taxa were presented, as published between August 2011 and June 2013:

*Salarchaeum* gen. nov. [Shimane *et al.*, *Int J Syst Evol Microbiol* **61** (2011), 2266–2270], with type species *Salarchaeum japonicum*. Recommended three-letter abbreviation: *Sar*.

*Salarchaeum japonicum* sp. nov. [Shimane *et al.*, *Int J Syst Evol Microbiol* **61** (2011), 2266–2270], with type strain JCM 16327^T^, CECT 7563^T^.

*Halobellus* gen. nov. [Cui *et al.*, *Int J Syst Evol Microbiol* **61** (2011), 2682–2689], with type species *Halobellus clavatus*. Recommended three-letter abbreviation: *Hbs*.

*Halobellus clavatus* [Cui *et al.*, *Int J Syst Evol Microbiol* **61** (2011), 2682–2689], with type strain CGMCC 1.10118^T^, JCM 16424^T^.

*Halorientalis* gen. nov. [Cui *et al.*, *Int J Syst Evol Microbiol* **61** (2011), 2682–2689], with type species *Halorientalis regularis*. Recommended three-letter abbreviation: *Hos*.

*Halorientalis regularis* sp. nov. [Cui *et al.*, *Int J Syst Evol Microbiol* **61** (2011), 2682–2689], with type strain CGMCC 1.10123^T^, JCM 16425^T^.

*Halogranum salarium* sp. nov. [Kim *et al.*, *Syst Appl Microbiol* **34** (2011), 576–580; Validation List no. 145, *Int J Syst Evol Microbiol* **62** (2012), 1017–1019], with type strain KCTC 4066^T^, DSM 23171^T^.

*Haloarchaeobius* gen. nov. [Makhdoumi-Kakhki *et al.*, *Int J Syst Evol Microbiol* **62** (2012), 1021–1026], with type species *Haloarchaeobius iranensis**.* Recommended three-letter abbreviation: *Hab*.

*Haloarchaeobius iranensis* sp. nov. [Makhdoumi-Kakhki *et al.*, *Int J Syst Evol Microbiol* **62** (2012), 1021–1026], with type strain IBRC-M 10013^T^, KCTC 4048^T^.

*Halomicrobium zhouii* sp. nov. [Yang & Cui, *Int J Syst Evol Microbiol* **62** (2012), 1235–1240], with type strain CGMCC 1.10457 ^T^, JCM 17095^T^.

*Halobellus limi* sp. nov. [Cui *et al.*, *Int J Syst Evol Microbiol* **62** (2012), 1307–1313], with type strain CGMCC 1.10331^T^, JCM 16811^T^.

*Halobellus salinus* sp. nov. [Cui *et al.*, *Int J Syst Evol Microbiol* **62** (2012), 1307–1313], with type strain CGMCC 1.10710^T^, JCM 14359^T^.

*Halovenus* gen. nov. [Makhdoumi-Kakhki *et al.*, *Int J Syst Evol Microbiol* **62** (2012), 1331–1336], with type species *Halovenus aranensis*. Recommended three-letter abbreviation: *Hvn*.

*Halovenus aranensis* sp. nov. [Makhdoumi-Kakhki *et al.*, *Int J Syst Evol Microbiol* **62** (2012), 1331–1336], with type strain IBRC-M 10015^T^, CGMCC 1.1101^T^.

*Halopenitus* gen. nov. [Amoozegar *et al.*, *Int J Syst Evol Microbiol* **62** (2012), 1932–1936], with type species *Halopenitus persicus*. Recommended three-letter abbreviation: *Hpt*.

*Halopenitus persicus* sp. nov. [Amoozegar *et al.*, *Int J Syst Evol Microbiol* **62** (2012), 1932–1936], with type strain IBRC 10041^T^, KCTC 4046^T^.

*Natrinema salaciae* sp. nov. [Albuquerque *et al.*, *Syst Appl Microbiol* **35** (2012), 368–373; Validation List no. 148, *Int J Syst Evol Microbiol* **62** (2012), 2549–2554], with type strain DSM 25055^T^, JCM 17869^T^, CECT 8172^T^.

*Natronococcus roseus* sp. nov. [Corral *et al.*, *Int J Syst Evol Microbiol* **63** (2013), 104–108], with type strain CECT 7984^T^, IBRC-M 10656^T^, JCM 17958^T^.

*Halobaculum magnesiiphilum* sp. nov. [Shimoshige *et al.*, *Int J Syst Evol Microbiol* **63** (2013), 861-866], with type strain JCM 17821^T^, KCTC 4100^T^.

*Natronoarchaeum philippinense* sp. nov. [Shimane *et al.*, *Int J Syst Evol Microbiol* **63** (2013), 920–924], with type strain JCM 16593^T^, CECT 7630^T^.

*Halarchaeum salinum* sp. nov. [Yamauchi *et al.*, *Int J Syst Evol Microbiol* **63** (2013), 1138–1142], with type strain JCM 16330^T^, CECT 7574^T^.

*Halopelagius fulvigenes* sp. nov. [Liu *et al.*, *Int J Syst Evol Microbiol* **63** (2013), 2192–2196], with type strain CCTCC AB 2010456^T^, JCM 17506^T^.

*Natronorubrum texcoconense* sp. nov. [Ruiz-Romero *et al.*, *Arch Microbiol* **195** (2013), 145–151], with type strain CECT 8067^T^, JCM 17497^T^.

The following names will appear in Validation List no. 152, to be published in July 2013:

*Halohasta* gen. nov. [Mou *et al.*, *Extremophiles* **16** (2012), 895–901], with type species *Halohasta litorea*. Recommended three-letter abbreviation: *Hht*.

*Halohasta litorea* sp. nov. [Mou *et al.*, *Extremophiles* **16** (2012), 895–901], with type strain CGMCC 1.10593^T^, JCM 17270^T^.

*Halohasta litchfieldiae* sp. nov. [Mou *et al.*, *Extremophiles* **16** (2012), 895–901], with type strain JCM 15066^T^, DSM 22187^T^, CCMCC 1.12394^T^.

*Halalkalicoccus paucihalophilus* sp. nov. [Liu *et al.*, *Antonie van Leeuwenhoek* **103** (2013), 1007–1014], with type strain JCM 17505^T^, CCTCC 2012803^T^.

As of 8 June 2013, descriptions of the following new taxa were in press in *Int J Syst Evol Microbiol*:

*Halarchaeum* *rubridurum* sp. nov. [Yamauchi *et al.*], with type strain JCM 16108^T^, CECT 27353^T^.

*Halopenitus* *malekzadehii* sp. nov. [Amoozegar *et al.*], with type strain IBRC-M 10418^T^, KCTC 4045^T^.

*Halopiger* *salifodinae* sp. nov. [Amoozegar *et al.*], with type strain JCM 18547^T^, CGMCC 1.12284^T^.

*Halomicroarcula* gen. nov. [Zhang *et al.*], with type species *Halomicroarcula pellucida*. As the authors did not propose a three-letter abbreviation, the joint subcommittee meeting recommended *Hma*.

*Halomicroarcula pellucida* sp. nov. [Zhang *et al.*], with type strain JCM 17820^T^, CECT 7537^T^.

*Halopelagius* *longus* sp. nov. [Zhang *et al.*], with type strain CGMCC 1.12397^T^, JCM 18758^T^.

*Halobellus* *inordinatus* sp. nov. [Qiu *et al.*], with type strain CGMCC 1.12120^T^, JCM 18648^T^.

*Natronobacterium* *texcoconense* sp. nov. [Ruiz-Romero *et al.*], with type strain CECT 8068^T^, JCM 17655^T^.

The following papers relevant to the taxonomy of *Halobacteriaceae* were published in *Int J Syst Evol Microbiol*:

A multilocus sequence analysis (MLSA) approach to *Halobacteriales* phylogeny and taxonomy [Papke *et al.*, *Int J Syst Evol Microbiol* **61** (2011), 2984–2995].

Gene orders in the upstream of 16S rRNA genes divide genera of the family *Halobacteriaceae* into two groups [Minegishi *et al.*, *Int J Syst Evol Microbiol* **62** (2012), 188–195].

Taxonomy of the family *Halobacteriaceae*: a paradigm for changing concepts in prokaryote systematics [Oren, *Int J Syst Evol Microbiol* **62** (2012), 263–271].

*Halobacterium piscisalsi* Yahai *et al.* 2008 is a later heterotypic synonym of *Halobacterium salinarum* Elazari-Volcani 1957 [Minegishi *et al.*, *Int J Syst Evol Microbiol* **62** (2012), 2160–2162].

The following names of members of the *Halobacteriaceae* were effectively but not validly published as of 8 June 2013:

*Salinarchaeum* gen. nov. [Cui *et al.*, *Extremophiles* **15** (2011), 625–631], with type species *Salinarchaeum laminariae*. Recommended three-letter abbreviation: *Saa*.

*Salinarchaeum laminariae* sp. nov. [Cui *et al.*, *Extremophiles* **15** (2011), 625–631], with type strain CGMCC 1.10590^T^, JCM 17267^T^.

*Halorubellus* gen. nov. [Cui *et al.*, *Syst Appl Microbiol* **35** (2012), 30–34], with type species *Halorubellus salinus*. Recommended three-letter abbreviation: *Hrb*.

*Halorubellus salinus* sp. nov. [Cui *et al.*, *Syst Appl Microbiol* **35** (2012), 30–34], with type strain CGMCC 1.10384^T^, JCM 17115^T^.

*Halorubellus litoreus* sp. nov. [Cui *et al.*, *Syst Appl Microbiol* **35** (2012), 30–34], with type strain CGMCC 1.10386^T^, JCM 17117^T^.

*Halarchaeum* *solikamskense* sp. nov. [Saralov *et al.*, *Microbiology* (English translation) **81** (2012), 589–595 (Russian original: 638–644)], with type strain VKPM B-11282^T^.

*Haloferax* *chudinovii* sp. nov. [Saralov *et al.*, *Extremophiles* **17** (2013), 499–504], with type strain VKPM B-11279^T^.

### Minute 8. Available genome sequences of *Halobacteriaceae*.

As of 8 June 2013, complete or draft genome sequences were available for type strains of the following species: *Haladaptatus paucihalophilus* (AEMG00000000.1; AQXI00000000.1); *Halalkalicoccus jeotgali* (CP002062.1–CP002068.1; AOHV00000000.1); *Haloarcula amylolytica* (AOLW00000000.1); *Haloarcula argentinensis* (AOLX00000000.1); *Haloarcula hispanica* (CP002921.1–CP002923.1); *Haloarcula japonica* (AOLY00000000.1); *Haloarcula marismortui* (AY596290.1–AY596298.1; PubSEED 272569.1); *Haloarcula vallismortis* (AOLQ00000000.1; PubSEED 662477.4); *Halobiforma lacisalsi* (AGFZ00000000.1; AOLZ00000000.1); *Halobiforma nitratireducens* (AOMA00000000.1); *Halococcus hamelinensis* (AJRK00000000.1; AOMB00000000.1); *Halococcus morrhuae* (AOMC00000000.1); *Halococcus saccharolyticus* (AOMD00000000.1); *Halococcus salifodinae* (AOME00000000.1); *Halococcus thailandensis* (AOMF00000000.1); *Haloferax denitrificans* (AOLP00000000.1; PubSEED 662478.4); *Haloferax alexandrinus* (AOLL00000000.1); *Haloferax elongans* (AOLK00000000.1); *Haloferax gibbonsii* (AOLJ00000000.1); *Haloferax larsenii* (AOLI00000000.1); *Haloferax lucentense* (AOLH00000000.1); *Haloferax mediterranei* (CP001868–CP001871; AOLO00000000.1; PubSEED 523841.6); *Haloferax mucosum* (AOLN00000000.1; PubSEED 662479.5); *Haloferax prahovense* (AOLG00000000.1); *Haloferax sulfurifontis* (AOLM00000000.1; PubSEED 662480.4); *Haloferax volcanii* (CP001953.1–CP001957.1; AOHU00000000.1)); *Halogeometricum borinquense* (CP01690.1–CP01695.1; AOHT00000000.1); *Halogranum salarium* (ALJD00000000.1); *Halomicrobium mukohataei* (CP001688.1; CP001689.1); *Halomicrobium katesii* (AQZY00000000.1); *Halopiger xanaduensis* (CP002839.1–CP002842.1); *Haloquadratum walsbyi* (FR746099.1–FR746102.1); *Halorhabdus tiamatea* (AFNT00000000.1); *Halorhabdus utahensis* (CP001687.1); *Halorubrum aidingense* (AOJI00000000.1); *Halorubrum arcis* (AOJJ00000000.1); *Halorubrum californiense* (AOJK00000000.1); *Halorubrum coriense* (AOJL00000000.1); *Halorubrum distributum* (AOJM00000000.1; AOJN00000000.1); *Halorubrum kocurii* (AOJH00000000.1); *Halorubrum lacusprofundi* (CP001365.1–CP001367.1); *Halorubrum litoreum* (AOJF00000000.1); *Halorubrum lipolyticum* (AOJG00000000.1); *Halorubrum saccharovorum* (AOJE00000000.1); *Halorubrum tebenquichense* (AOJD00000000.1); *Halorubrum terrestre* (AOIW00000000.1); *Haloterrigena limicola* (AOIT00000000.1); *Haloterrigena salina* (AOIS00000000.1); *Haloterrigena thermotolerans* (AOIR00000000.1); *Haloterrigena turkmenica* (CP001860.1–CP001866.1); *Halovivax asiaticus* (AOIQ00000000.1); *Halovivax ruber* (CP003050.1); *Natrialba aegyptia* (AOIP00000000.1); *Natrialba asiatica* (AOIO00000000.1); *Natrialba chahannaoensis* (AOIN00000000.1); *Natrialba hulunbeirensis* (AOIM00000000.1); *Natrialba magadii* (CP001932.1–CP001935.1; AOHS00000000.1); *Natrialba taiwanensis* (AOIL00000000.1); *Natrinema altunense* (AOIK00000000.1); *Natrinema gari* (AOIJ00000000.1); *Natrinema pallidum* (AOII00000000.1); *Natrinema pellirubrum* (CP003372.1–CP003374.1; AOIE00000000.1); *Natrinema versiforme* (AOID00000000.1); *Natronobacterium gregoryi* (CP003377.1; AOIC00000000.1); *Natronococcus amylolyticus* (AOIB00000000.1); *Natronococcus jeotgali* (AOIA00000000.1); *Natronococcus occultus* (CP003929.1-CP003931.1); *Natronomonas moolapensis* (HF582854.1); *Natronomonas pharaonis* (CR936257.1–CR936259.1).

Additional sequenced strains are ‘Haloarcula californiae’ ATCC 33799 (AOLS00000000.1; PubSEED 662475.4); ‘Haloarcula sinaiiensis’ ATCC 33800 (AOLR00000000.1; PubSEED 662476.5); *Halobacterium* sp. DL1 (AGIR00000000.1); *Halobacterium* sp. DL31 (CP002988.1-CP002990.1); *Halobacterium* sp. NRC-1, ATCC 700922 (AE004437.1, AF016485.1, AE004438.1); *Halobacterium salinarum* R1, DSM 671 (AM774415.1–AM774419.1); *Haloferax* sp. ATCC BAA-644 (AOLF00000000.1); *Haloferax* sp. ATCC BAA-645 (AOLE00000000.1); *Haloferax* sp. ATCC BAA-646 (AOLD00000000.1); *Haloferax* sp. BAB2207 (ANPG00000000.1); *Haloquadratum walsbyi* DSM 16790 (AM180088.1, AM180089.1); ‘Halorubrum hochstenium’ ATCC 700873 (AOJO00000000.1); *Halorubrum* sp. T3 (ALWQ00000000.1); *Natrinema* sp. J7-2 (CP003412.1, CP003413.1).

### Minute 9. New taxa within the family *Halomonadaceae*.

As of June 2013 the family *Halomonadaceae* contained 10 genera (name and number of species with validly published names): *Halomonas* (76); *Aidingimonas* (1); *Carnimonas* (1); *Chromohalobacter* (8); *Cobetia* (5); *Halotalea* (1); *Kushneria* (5); *Modicisalibacter* (1); *Salinicola* (3); *Zymobacter* (1), total 102 species. Reports on the following new taxa and reclassification of existing taxa were presented, as published between August 2011 and June 2013:

*Halomonas sinaiensis* sp. nov. [Romano *et al.*, *Extremophiles* **11** (2007), 789–796; Validation List no. 141, *Int J Syst Evol Microbiol* **61** (2011), 2025–2026], with type strain DSM 18067^T^, ATCC BAA-1308^T^.

*Halomonas stenophila* sp. nov. [Llamas *et al.*, *Int J Syst Evol Microbiol* **61** (2011), 2508–2514], with type strain CECT 7744^T^, LMG 25812^T^.

*Halomonas jeotgali* sp. nov. [Kim *et al.*, *J Microbiol* **48** (2010), 404–410; Validation List no. 142, *Int J Syst Evol Microbiol* **61** (2011), 2563–2565], with type strain JCM 15645^T^, KCTC 22487^T^.

*Halomonas alkaliantarctica* sp. nov. [Poli *et al.*, *Syst Appl Microbiol* **30** (2007), 31–38; Validation List no. 142, *Int J Syst Evol Microbiol* **61** (2011), 2563–2565], with type strain ATCC BAA-848^T^, DSM 15686^T^.

*Halomonas rifensis* sp. nov. [Amjres *et al.*, *Int J Syst Evol Microbiol* **61** (2011), 2600–2605], with type strain CECT 7698^T^, LMG 25695^T^.

*Halomonas xianhensis* sp. nov. [Zhao *et al.*, *Int J Syst Evol Microbiol* **62** (2012), 173–178], with type strain CGMCC 1.6848^T^, JCM 14849^T^.

*Halomonas flava* sp. nov. [Chen *et al.*, *Antonie van Leeuwenhoek* **100** (2011), 365–373; Validation List no. 147, *Int J Syst Evol Microbiol* **62** (2012), 2045–2047], with type strain CCTCC AB 02010382^T^, KCTC 23356^T^.

*Halomonas qijiaojingensis* sp. nov. [Chen *et al.*, *Antonie van Leeuwenhoek* **100** (2011), 365–373; Validation List no. 147, *Int J Syst Evol Microbiol* **62** (2012), 2045–2047], with type strain CCTCC AB 02008133^T^, KCTC 22228^T^.

*Halomonas ramblicola* sp. nov. [Luque *et al.*, *Int J Syst Evol Microbiol* **62** (2012), 2903–2909], with type strain CECT 7896^T^, LMG 26647^T^.

*Halomonas beimenensis* sp. nov. [Wang *et al.*, *Int J Syst Evol Microbiol* **62** (2012), 3013–3017], with type strain BCRC 17999^T^, KCTC 22876^T^, JCM 16084^T^.

*Halomonas smyrnensis* sp. nov. [Poli *et al.*, *Int J Syst Evol Microbiol* **63** (2013), 10–18], with type strain DSM 21644^T^, JCM 15723^T^.

*Cobetia amphilecti* sp. nov. [Romanenko *et al.*, *Int J Syst Evol Microbiol* **63** (2013), 288–297], with type strain NRIC 0815^T^, CCUG 49560^T^.

*Cobetia litoralis* sp. nov. [Romanenko *et al.*, *Int J Syst Evol Microbiol* **63** (2013), 288–297], with type strain NRIC 0814^T^, CCUG 49563^T^.

*Cobetia pacifica* sp. nov. [Romanenko *et al.*, *Int J Syst Evol Microbiol* **63** (2013), 288–297], with type strain NRIC 0813^T^, CCUG 49562^T^.

*Halomonas cibimaris* sp. nov. [Jeong *et al.*, *Antonie van Leeuwenhoek* **103** (2013), 503–512; Validation List no. 151, *Int J Syst Evol Microbiol* **63** (2012), 1577–1580], with type strain KACC 14932^T^, JCM 16914^T^.

An updated list of taxa will be uploaded on the home page of the subcommittee on the ICSP website.

The following papers relevant to the taxonomy of *Halomonadaceae* were published in *Int J Syst Evol Microbiol*:

Classification of *Halomonas halodurans* as a later heterotypic synonym of *Cobetia marina*. [Romanenko *et al.*, *Int J Syst Evol Microbiol* **63** (2013), 288–297].

Emended description of *Cobetia* [Romanenko *et al.*, *Int J Syst Evol Microbiol* **63** (2013), 288–297].

Emended description of *Cobetia marina* [Romanenko *et al.*, *Int J Syst Evol Microbiol* **63** (2013), 288–297].

Multilocus sequence analysis (MLSA) of the family *Halomonadaceae* [de la Haba *et al.*, *Int J Syst Evol Microbiol* **62** (2012), 520–538].

The following names of members of the *Halomonadaceae* were effectively but not validly published as of 8 June 2013:

*Halomonas* *aidingensis* sp. nov. [Liu *et al.*, *Antonie van Leeuwenhoek* **99** (2011), 663–670], with type strain CGMCC 1.10191^T^, NBRC 106173^T^.

*Salinicola* *zeshunii* sp. nov. [Cao *et al.*, *Curr Microbiol* **66** (2013), 192–196], with type strain KACC 16602^T^, CCTCC AB 2012912^T^.

*Halomonas nanhaiensis* sp. nov. [Long *et al.*, *Antonie van Leeuwenhoek* **103** (2013), 997–1005], with type strain JCM 18142^T^, CCTCC AB 2012911^T^.

*Salinicola* *peritrichatus* sp. nov. [Huo *et al.*, *Antonie van Leeuwenhoek* published online: 23 April 2013 (doi: 10.1007/s10482-013-9925-1)], with type strain CGMCC 1.12381^T^, JCM 18795 ^T^.

*Halomonas* *socia* sp. nov. [Cao *et al.*, *Extremophiles* in press] with type strain CCTCC AB 2011033^T^, KCTC 23671^T^.

### Minute 10. Available genome sequences of *Halomonadaceae*.

As of 8 June 2013, complete or draft genome sequences were available for type strains of the following species: *Chromohalobacter salexigens* (CP000285.1); *Halomonas anticariensis* (ASTJ00000000.1); *Halomonas boliviensis* (AGQZ00000000.1); *Halomonas elongata* (FN869568.1); *Halomonas jeotgali* (AMQY00000000.1); *Halomonas lutea* (ARKK00000000.1); *Halomonas smyrnensis* (AJKS00000000.2); *Halomonas stevensii* (AJTS00000000.1); *Halomonas titanicae* (AOPO00000000.1); *Halomonas zhanjiangensis* (ARIT00000000.1); *Kushneria aurantia* (ARNK00000000.1).

Additional sequenced strains are *Halomonas* sp. GFAJ-1 (AHBC00000000.1); *Halomonas* sp. HAL1 (AGIB00000000.1); *Halomonas* sp. KM-1 (BAEU00000000.1); *Halomonas* sp. TD01 (AFQW00000000.1).

### Minute 11. Recommended minimal standards.

The recommended minimal standards for describing new taxa of the family *Halobacteriaceae* were published in 1997 [Oren *et al.*, *Int J Syst Evol Microbiol* **47** (1997), 233–238], and the recommended minimal standards for describing new taxa of the family *Halomonadaceae* were published in 2007 [Arahal *et al.*, *Int J Syst Evol Microbiol* **57** (2007), 2436–2446]. The current relevance of these minimal standards was discussed. It was felt that the current minimal standards documents are still adequate in combination with Notes on the characterization of prokaryote strains for taxonomic purposes [Tindall *et al.*, *Int J Syst Evol Microbiol* **60** (2010), 249–266], but the following additions were suggested. 1. It was stressed that it is highly desirable to base the description of new species on more than one strain. 2. A good database of sequences of suitable genes for multilocus sequence analysis is now available (see Minute 12), and inclusion of multilocus sequence analysis in the minimal standards is highly desirable. 3. Fatty acid analysis should be added as required rather than recommended in the recommended standards for describing new taxa of the family *Halomonadaceae*.

### Minute 12. Multilocus sequence analysis of *Halobacteriaceae*.

Satisfactory sets of genes for multilocus sequence analysis have now been proposed for the *Halobacteriaceae* [Papke *et al.*, *Int J Syst Evol Microbiol* **61** (2011), 2984–2995] and for the *Halomonadaceae* [de la Haba *et al.*, *Int J Syst Evol Microbiol* **62** (2012), 520–538]. It is therefore recommended in addition to the 16S rRNA gene(s), descriptions of novel species should include sequence information on the following genes: *atpB*, *EF-2*, *glnA*, *ppsA* and *rpoB* for members of the *Halobacteriaceae*, and *gyrB* and *rpoD* for members of the *Halomonadaceae*.

### Minute 13. Next meeting of the subcommittees.

The next meetings of both subcommittees will be held in association with the International Union of Microbiological Societies Congresses, Montreal, Canada, in July–August 2014.

### Minute 14. Any other business.

There was no other business.

### Minute 15. Adjournment.

The meeting was adjourned at 20 : 10 on 24 June 2013.

